# Dexamethasone Prophylaxis in Pediatric Open Heart Surgery Is Associated with Increased Blood Long Pentraxin PTX3: Potential Clinical Implications

**DOI:** 10.1155/2011/730828

**Published:** 2011-07-09

**Authors:** Franco Lerzo, Giuseppe Peri, Andrea Doni, Paola Bocca, Fabio Morandi, Angela Pistorio, Anna Maria Carleo, Alberto Mantovani, Vito Pistoia, Ignazia Prigione

**Affiliations:** ^1^Division of Cardiovascular Surgery, G. Gaslini Institute, IRCCS, Largo G. Gaslini 5, 16148 Genoa, Italy; ^2^Department of Immunology and Inflammation, Istituto Clinico Humanitas, IRCCS, Via Manzoni 56, 20089 Rozzano, Milan, Italy; ^3^Laboratory of Oncology, G. Gaslini Institute, IRCCS, Largo G. Gaslini 5, 16148 Genoa, Italy; ^4^Clinical Epidemiology and Biostatistics Unit, G. Gaslini Institute, IRCCS, Largo G. Gaslini 5, 16148 Genoa, Italy; ^5^Dipartimento di Medicina Traslazionale, University of Milan, Via Manzoni 56, 20089 Rozzano, Milan, Italy

## Abstract

Glucocorticoid administration before cardiopulmonary bypass (CPB) can reduce the systemic inflammatory response and improve clinical outcome. Long pentraxin PTX3 is a novel inflammatory parameter that could play a protective cardiovascular role by regulating inflammation. Twenty-nine children undergoing open heart surgery were enrolled in the study. Fourteen received dexamethasone (1st dose 1.5 mg/Kg i.v. or i.m. the evening before surgery; 2nd dose 1.5 mg/kg i.v. before starting bypass) and fifteen children served as control. Blood PTX3, short pentraxin C-reactive protein (CRP), interleukin-1 receptor II (IL-1RII), fibrinogen and partial thromboplastin time (PTT) were assayed at different times. PTX3 levels significantly increased during CPB in dexamethasone-treated (+D) and dexamethasone-untreated (−D) subjects, but were significantly higher in +D than −D patients. CRP levels significantly increased both in +D and −D patients in the postoperative days, with values significantly higher in −D than +D patients. Fibrinogen and PTT values were significantly higher in −D than +D patients in the 1st postoperative day. IL-1RII plasma levels increased in the postoperative period in both groups. Dexamethasone prophylaxis in pediatric patients undergoing CPB for cardiac surgery is associated with a significant increase of blood PTX3 that could contribute to decreasing inflammatory parameters and improving patient clinical outcome.

## 1. Introduction

Cardiopulmonary bypass (CPB) procedures during cardiac surgery produce systemic effects, especially in younger children. The contact of cellular and humoral blood components with biocompatible synthetic material of extracorporeal circuit provokes a systemic inflammatory response (SIR) involving leukocyte and endothelial cell activation and cytokine release, which leads to myocardial, renal, and pulmonary dysfunctions and has a negative impact on the postoperative clinical course [1, 2].

Different anti-inflammatory strategies have been used to minimize the CPB-related organ dysfunctions, including steroid prophylaxis with different schedules of administration. Although glucocorticoid (GC) administration before CPB has been found to result in a reduced inflammatory response, the benefits of glucocorticoids on clinical outcome of adult and pediatric patients are debated [3–9].

Pentraxins are a superfamily of proteins belonging to the humoral arm of innate immunity and including the classical short pentraxins (C-reactive protein (CRP) and serum amyloid P component in human and mice, resp.) and long pentraxins [10, 11].

CRP, prototype of the short pentraxin family, is an acute-phase protein in humans. It is produced in the liver in response to inflammatory signals, predominantly IL-6, it interacts with different ligands, and it is involved in innate resistance to different pathogens. 

Long Pentraxin PTX3 is a novel inflammatory marker, prototype of the long pentraxin family, produced by innate immune cells and vascular cells in response to proinflammatory signals [10, 12]. PTX3 is a multifunctional protein and plays complex, nonredundant roles in vivo, recognizing a diverse range of pathogens, modulating complement activity, and facilitating pathogen recognition by macrophages and dendritic cells (DCs). Several evidences link PTX3 and cardiovascular diseases: PTX3 production by smooth muscle cells stimulated by atherogenic LDL, localization in atherosclerotic lesions, and high expression level observed in the heart during inflammatory reactions [13, 14]. PTX3 levels increase rapidly in patients with acute myocardial infarction (AMI), emerging as the only independent predictor of mortality [15]. In addition PTX3 plasma levels are elevated in patients with unstable angina and in patients undergoing stenting, suggesting that PTX3 is a candidate for being new prognostic marker in ischemic heart disorders [16–18]. However, beside the role of PTX3 as a cardiovascular biomarker associated with inflammatory reactions, recent in vivo and in vitro data point to a protective cardiovascular role of PTX3 through a regulatory role on inflammation [19, 20]. In this respect, increased levels of PTX3 could reflect a protective response of the host.

Soluble cytokine receptor release could represent a mechanism to counterbalance inflammatory responses. IL-1 is a key cytokine in inflammation and represents an important target of GC-mediated immunosuppressive activities. GC suppresses IL-1 production but augments cell surface expression of IL-1 receptor (R)II with consequently enhanced release of the soluble form of the receptor itself [21, 22]. IL-1 RII has no signalling properties, acts as a “decoy” target for IL-1, binding with high affinity to IL-1 and preventing its binding to the signalling IL-1RI [23].

Here we have analysed the influence of dexamethasone prophylaxis in pediatric patients undergoing CPB on blood levels of PTX3, IL-1 RII, and other inflammatory parameters.

## 2. Patients and Methods

### 2.1. Patients

Twenty-nine children admitted to the Cardiovascular Surgery Unit of the G. Gaslini Institute were enrolled in the study (Table 1). Inclusion criteria were body weight <10 Kg and type of surgery (biventricular corrections), excluding neonates and all residual intracardiac shunts that could prevent the analysis of pulmonary oxygen exchanges. This study protocol has been approved by the Ethical Committee of the G. Gaslini Institute in Genoa, Italy. 

Patients were randomized into two groups: (a) +D: 14 children receiving prophylaxis with dexamethasone; (b) −D: 15 controls. Group +D patients received two doses of 1.5 mg/Kg dexamethasone i.v. or i.m.: the first dose at the evening before surgery, the second dose 30 minutes before starting CPB. All patients in both groups were treated with the same protocols for anaesthesia and extracorporeal perfusion. Blood samples were collected preoperatively before steroid administration (*T*1), 10 min after the starting of CPB (*T*2), after aortic cross-clamping release (*T*3), at the end of CPB (*T*4), and on the 1st (*T*5) and 2nd (*T*6) postoperative day. The method used to assign interventions to trial participants was a simple random assignment with an allocation sequence generated by an automatic routine (with the software SPSS), with an allocation ratio of 1 : 1. The two patient groups were homogeneous in terms of gender, age, and weight (Table 1).

Patient clinical evaluation included duration of CPB and aortic declamping, duration of mechanical ventilation, duration of stay in Intensive Care Unit (ICU), and postoperative blood loss and alveolar-arterial oxygen differences at T5 (Table 1). No relevant complications were observed in the perioperative course.

### 2.2. Assays for PTX3, IL-1 RII, CRP, Fibrinogen, and PTT

Blood samples were collected at the indicated time points (see above) in tubes containing ethylenediaminetetraacetic acid. Plasma was obtained by sample centrifugation and stored at −80°C until use. PTX3 plasma levels were measured by an in-house ELISA as previously described [24, 25]. Detection limit is 100 pg/mL and interassay variability is 8–10%. IL-1RII was measured by sandwich ELISA using the anti-IL-1RII monoclonal antibody 8.5 and an anti-IL-1RII polyclonal antibody, both of which were generated by some of us [21]. The lowest detection limit of this assay was 20 pg/mL.

CRP was measured by immunoturbidimetric assay (Roche Diagnostic S.p.A Milano, Italia). Fibrinogen and PTT were measured by photometric and turbidimetric detection systems (BCS-XP, Siemens Healthcare Diagnostic, Deerfield, Ill, USA).

### 2.3. Statistical Analysis

Descriptive analyses were firstly performed; qualitative data were reported in terms of absolute frequencies and percentages, and quantitative data were reported as median values with first and third quartiles (1st–3rd q). Comparison of qualitative data between two groups of patients (treated versus untreated) was performed by the chi-square test; comparison of quantitative data between two groups of patients was performed by the Mann-Whitney *U* test.

The comparison of quantitative data at different time points was evaluated by the nonparametric analysis of variance (Friedman's test), and the post hoc analysis was made by the Wilcoxon test adjusted with Bonferroni's correction.

In order to evaluate differences between quantitative data at each time point between the two groups of patients (treated versus untreated), the Mann-Whitney *U* test adjusted with the Bonferroni's correction was applied. In the figure data were presented as median values with first and third quartiles.

All the statistical tests were two sided and a *P* value less than  .05 was considered as statistically significant. The software “Statistica” (StatSoft Co., Tulsa, OKla, USA) was used for all the statistical analyses.

## 3. Results

### 3.1. Effect of Dexamethasone Prophylaxis on Blood Levels of Pentraxins (PTX3/CRP) and Other Inflammatory Parameters

**Figure 1:**
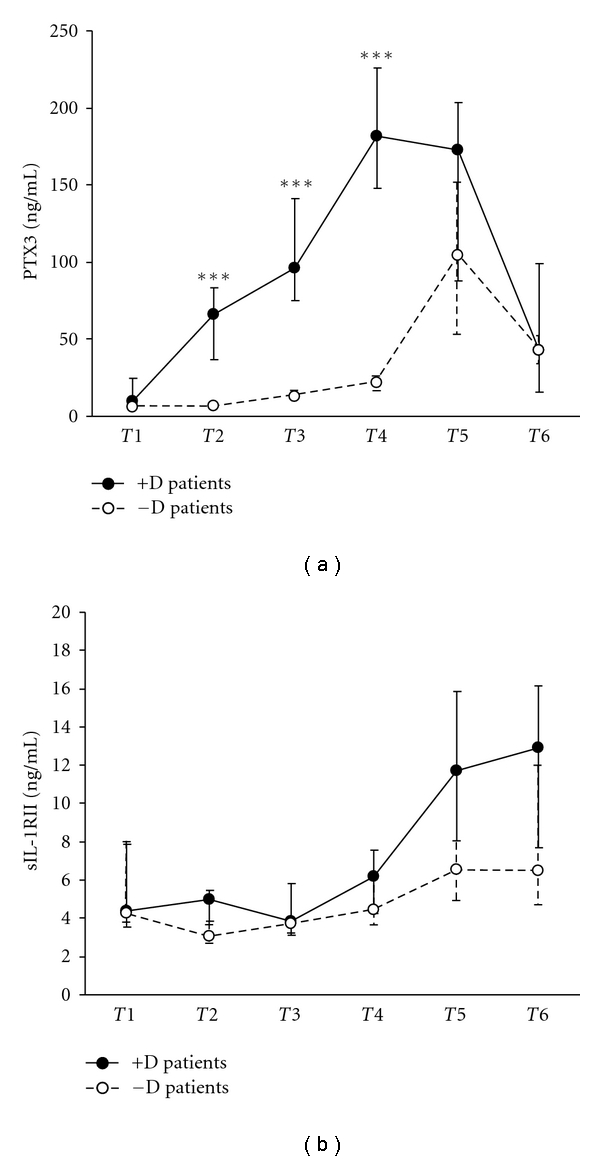
Plasma levels of PTX3 (a) and soluble IL1 receptor II (b) in pediatric patients undergoing CPB, either treated or untreated with dexamethasone. Values are expressed as medians and 1st and 3rd quartile. Time of blood sampling: *T*1, preoperative, before steroid administration; *T*2, 10 min after CPB; *T*3, after aortic declamping; *T*4, at the end of CPB; *T*5, 1st postoperative day; *T*6, 2nd postoperative day.***P < 0.001.

PTX3 levels were assayed in plasma samples collected at different times in 12 patients from group +D and 12 from group −D. When compared with baseline levels observed at *T*1 (median 5.87 ng/mL), PTX3 concentration in patients from group −D increased significantly during CPB at *T*3 (median 12.39 ng/mL, *P* = 0.0184),*T*4 (median 21.76 ng/mL, *P* = 0.0110), *T*5 (median 104.61 ng/mL, *P* = 0.0110), and *T*6 (median 42.44 ng/mL, *P* = 0.0110). PTX3 plasma levels at *T*2 did not differ significantly from those detected at *T*1 (Figure 1(a)).

Plasma levels of PTX3 in group +D patients, when compared with plasma concentrations detected in *T*1 (9.72 ng/mL), increased significantly at *T*2 (median 66.37 ng/mL, *P* = 0.0235), *T*3 (96.04 ng/mL, *P* = 0.0110), *T*4 (181.42 ng/mL, *P* = 0.0110), and *T*5 (172.7 ng/mL, *P* = 0.0110). PTX3 levels at *T*6 in group +D patients did not significantly differ from those detected at *T*1 (Figure 1(a)).

Plasma concentrations of PTX3 during CPB in +D patients were significantly higher than those detected in −D patients at *T*2, *T*3, and *T*4 (*P* = 0.0002), but did not differ at *T*1, *T*5, and *T*6 (Figure 1(a)).

Plasma levels of soluble IL-1RII were assayed during CPB in 12 −D and 8 +D patients (Figure 1(b)). Increased IL-1RII levels were detected in both groups at *T*5 and *T*6 (*T*5: 6.35 ng/mL, *T*6: 6.47 ng/mL median values in −D; *T*5: 11.72 ng/mL, *T*6: 12.9 ng/mL median values in +D) in comparison with baseline values (4.27 ng/mL and 4.38 ng/mL median values in −D and +D, resp.) (Figure 1(b)). These results showed a trend towards, but did not reach, statistical significance possibly due to the limited sample analyzed. Similarly, IL-1RII levels at *T*5 and *T*6 in group +D, although higher, did not significantly differ from those observed in group −D at the same time points.

**Figure 2:**
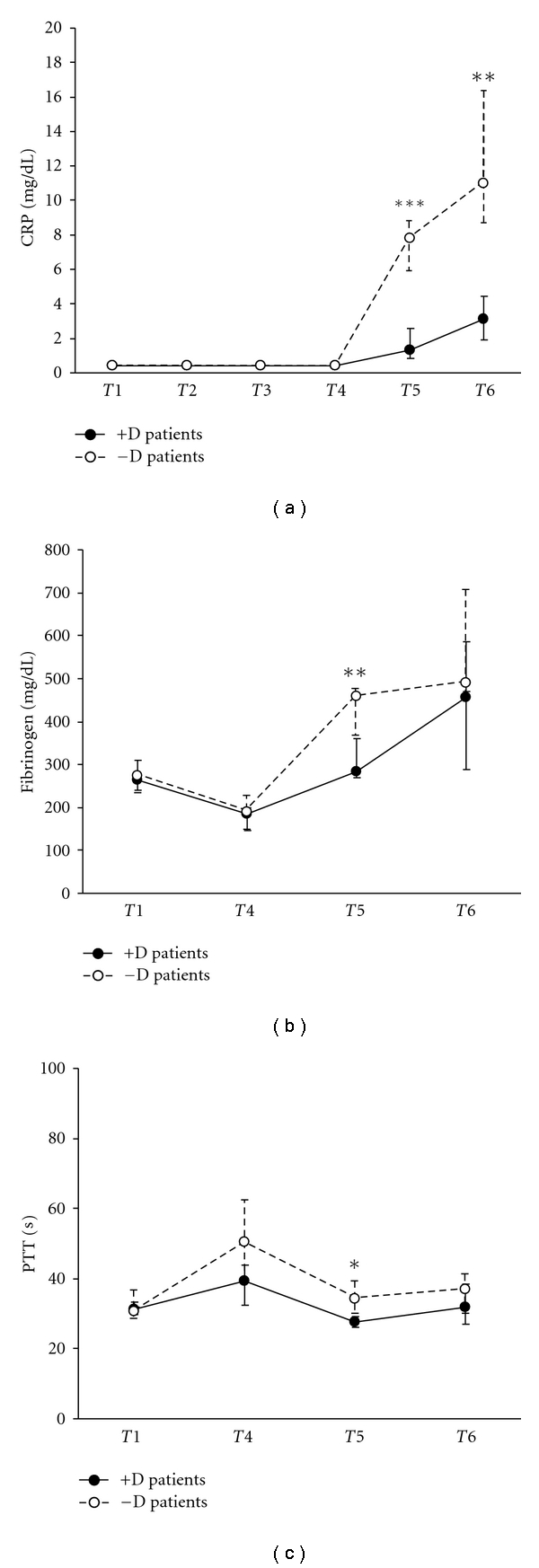
Plasma levels of CRP (a) and fibrinogen (b), and PTT values (c) in children undergoing CPB, either treated or untreated with dexamethasone. Values are expressed as medians and 1st and 3rd quartile. Time points are defined in Figure 1. ***P < 0.001; **P < 0.01; *P < 0.05.

Short pentraxin CRP plasma levels were assayed during CPB in 14 −D and 13 +D patients. As shown in Figure 2(a), a significant increase of CRP was detected both in −D and +D patients at *T*5 (−D 7.8 mg/dL, +D 1.3 mg/dL median values; *P* = 0.0049 and *P* = 0.0073, resp.) and *T*6 (−D 11 mg/dL, +D 3.1 mg/dL median values; *P* = 0.0049 and *P* = 0.0073, resp.) as compared to the baseline levels (0.4 mg/dL median value for both groups). It is of note that CRP values in −D patients at *T*5 and *T*6 were significantly higher than those detected in +D patients (*T*5 *P* = 0.0002; *T*6 *P* = 0.0034).

Finally, fibrinogen plasma concentrations and PTT were tested at *T*1, *T*4, *T*5, and *T*6 in 11 patients. Consistent with the CRP results, significantly decreased fibrinogen concentrations (−D 460 mg/dL, +D 284 mg/dL; *P* = 0.004) and PTT (−D 34.3 sec, +D 27.6 sec; *P* = 0.025) were detected in +D versus −D patients at T5 (Figures 2(b) and 2(c), resp.).

## 4. Discussion

This study demonstrates for the first time that dexamethasone prophylaxis in pediatric patients undergoing cardiopulmonary bypass for heart surgery is associated with significantly increased plasma levels of the long pentraxin PTX3 at different time points. This result was obtained from the study of a homogenous patient group selected in the frame of a clinical trial. Evidence for the potent anti-inflammatory effects of dexamethasone was obtained from laboratory studies; patient clinical evaluation pointed to a beneficial effect of steroid prophylaxis on the postoperative outcome which however did not reach statistical significance.

 Steroid prophylaxis during CPB on inflammation and clinical postoperative recovery has been investigated in different studies with opposite results, that is, beneficial versus irrelevant effects. These discrepancies may be related to the age of patients enrolled in these studies and to dosage, timing and the type of steroid administered [3–9].

PTX3 was found to be increased also in control patients not receiving dexamethasone. However, (i) PTX3 levels were significantly higher in dexamethasone-treated versus dexamethasone-untreated patients and (ii) increased and returned to baseline values earlier in the former than in latter group.

Plasma concentrations of short pentraxin CRP, which represents a reliable inflammatory marker, were similar in +D-treated versus −D patients until *T*4, when PTX3 levels were significantly higher in the former than the latter group. CRP plasma levels were increased in −D versus +D patients at *T*5 and *T*6, when PTX3 levels were not statistically different in the two groups. These latter results may be explained on the ground of the potent anti-inflammatory activity of dexamethasone. Superimposable behaviour was observed for fibrinogen plasma levels and PTT, both of which were significantly higher in −D versus +D patients at *T*5 only.

IL1-RII is an IL-1 decoy receptor endowed with anti-inflammatory activity. We found increased serum levels of IL-1RII in glucocorticoid-treated patients which however did not reach statistical significance. 

Dexamethasone was previously found to inhibit the lipopolysaccharide-induced PTX3 production in myeloid DC [26]. In contrast, in fibroblasts and endothelial cells, dexamethasone alone induced and, under inflammatory conditions, enhanced PTX3 production. The divergent effect of glucocorticoid on PTX3 regulation is likely due to differences in the functionality of their receptor in different cell populations. Glucocorticoid receptor could act as ligand-dependent transcription factor through direct DNA binding (dimerization-dependent), or as gene transcription repressor through protein-protein interference with the action of another signalling pathway (dimerization-independent) [27, 28]. In nonhaematopoietic cells the stimulation of PTX3 gene expression and production is dimerization dependent; on the contrary, suppression of PTX3 production in cells of haematopoietic origin is dimerization independent and mediated by interference with other signalling pathways, most likely the NF-kB and AP-1 pathways [26]. Thus the increased PTX3 levels observed in the present investigation could be in part due to the regulatory role exerted by glucocorticoids on PTX3 expression.

A further attractive candidate is IL-10, an anti-inflammatory cytokine that is known to increase in sera from patients receiving methylprednisolone during cardiopulmonary bypass [5]. IL-10 is a mild inducer of PTX3 expression in DC and monocytes [29]. In this respect, we have observed that plasma IL-10 concentrations, tested at *T*3 and *T*4, were augmented in some +D versus −D patients (data not shown). These preliminary findings warrant further investigation.

Recent observations *in vivo* in the mouse demonstrate that PTX3 can modulate neutrophils recruitment at inflamed site through the interaction with P-selectin, a molecule involved in the early steps of leukocyte recruitment [30] These data provide a first description of the mechanisms underlining the regulatory role on inflammation exerted by PTX3.

In conclusion, in this study, dexamethasone prophylaxis did not significantly affect the postoperative course although significantly inhibited the inflammatory response. In particular, PTX3 increase in patients treated with dexamethasone may be involved in downregulation of inflammatory parameters and represent a mechanism of vascular endothelium protection.

## Figures and Tables

**Table 1:** Demographic and clinical characteristics of the patients.

	Treated (+Dexamethasone)	Untreated (−Dexamethasone)	*P*
	No. (%)	No. (%)	
Gender: females	5/14 (35.7%)	9/15 (60%)	0.19 ^a^

	Median (1st–3rd q)	Median (1st–3rd q)	

Age (months)	9 (5–14)	6 (3–18)	0.30
Weight (Kg)	7.9 (6.1–8.1)	5.6 (4.0–9.8)	0.11

CPB (minutes)	95 (83–112)	93 (81–122)	0.84
AXT (minutes)	64 (59–76)	62 (46–73)	0.49
Mechanical ventilation (hours)	16.5 (9–48)	24 (16–32)	0.26
ICU stay (days)	1 (1–3)	2 (1.5–3)	0.10
Blood loss at T5 (ml/Kg/hr)	0.8 (0.7–0.9)	0.9 (0.7–1.5)	0.38
Alveolar-arteriolar oxygen difference at T5 (mmHg)	219 (158–316)	293 (152–393)	0.58

CPB: cardiopulmonary bypass; AXT: duration of aortic clamping; ICU: intensive care unit;  ^a^Chi-square test.

All *P* values refer to Mann-Whitney *U* test unless otherwise specified.
